# Atto-Foxes and Other Minutiae

**DOI:** 10.1007/s11538-021-00936-x

**Published:** 2021-08-31

**Authors:** A. C. Fowler

**Affiliations:** 1 MACSI, University of Limerick, Limerick, Ireland; 2 OCIAM, University of Oxford, Oxford, UK

**Keywords:** Atto-foxes, Boom-and-bust, Extinction, Stochastic logistic model, Frogspawn

## Abstract

This paper addresses the problem of extinction in continuous models of population dynamics associated with small numbers of individuals. We begin with an extended discussion of extinction in the particular case of a stochastic logistic model, and how it relates to the corresponding continuous model. Two examples of ‘small number dynamics’ are then considered. The first is what Mollison calls the ‘atto-fox’ problem (in a model of fox rabies), referring to the problematic theoretical occurrence of a predicted rabid fox density of $10^{-18}$ (*atto*-) per square kilometre. The second is how the production of large numbers of eggs by an individual can reliably lead to the eventual survival of a handful of adults, as it would seem that extinction then becomes a likely possibility. We describe the occurrence of the atto-fox problem in other contexts, such as the microbial ‘yocto-cell’ problem, and we suggest that the modelling resolution is to allow for the existence of a reservoir for the extinctively challenged individuals. This is functionally similar to the concept of a ‘refuge’ in predator–prey systems and represents a state for the individuals in which they are immune from destruction. For what I call the ‘frogspawn’ problem, where only a few individuals survive to adulthood from a large number of eggs, we provide a simple explanation based on a Holling type 3 response and elaborate it by means of a suitable nonlinear age-structured model.

## Preamble

It is a privilege to present this paper in a special issue of the journal in honour of Jim Murray’s 90th birthday in January 2021. Jim is of course a legend in the field of mathematical biology. He was also my undergraduate tutor at Corpus Christi College, Oxford, whom I first encountered by candlelight on a murky December evening 50 years ago last year (2020). I was asked recently by his contemporary Fellow Brian Harrison whether Jim had been the inspiration behind my own development as an applied mathematician. I suspect the answer is no, it would have happened anyway, but it is one of those unanswerable questions. What is undoubtedly true is that I have followed his inspirational mantra in aiming to be a genuinely applied mathematician. Jim is a marvellously interesting man, as anyone who reads his enthralling memoir (Murray 2018) will know. Jim and I each grew a beard over the same summer, and of our many overlapping interests—old furniture, home improvement, mediaeval literature, carpentry, red wine—it is perhaps the only thing we have in common where I could lay claim to parity.

## Introduction

In a recent fascinating conversation with the ecologist Yvonne Buckley, of Trinity College, Dublin, we touched on a number of issues concerning population dynamics which have puzzled me for some time. In this paper I want to draw these conundrums together and offer some palliative solutions.

The central theme is the use of continuous population models in circumstances where the population levels become extremely low. A continuous population model relies on the assumption that the population size varies continuously in time. This requires, for two reasons, that the population size be large. Firstly, because the actual discrete changes in integer numbers need to be viewed as infinitesimal changes, and secondly, because actual finite time gestation periods can only be interpreted as continuous in time if the large population can be taken as a realisation of an evolving probability density. Continuous population models are in particular subject to criticism when they indicate very low population levels, and in this circumstance discrete and/or stochastic models may be preferable (Durrett and Levin 1994).

The issue is simply illustrated by a linear birth-death process (Bartlett 1960) in which, if the population is of size *n*, individuals have probability $\lambda _n\,\delta t$ of giving birth in a time interval $\delta t$ and equivalent probability of dying of $\mu _n\,\delta t$. If $p_n(t)$ is the probability that the population is of size *n* at time *t*, then it is straightforward to show that

1.1 $$\begin{aligned} {\dot{p}}_n=(n-1)\lambda _{n-1} p_{n-1}-n(\lambda _n+\mu _n)p_n+(n+1)\mu _{n+1} p_{n+1}; \end{aligned}$$

this applies for $n\ge 0$, providing we define $p_{-1}=0$. Note that $p_0$ is the probability of extinction at time *t*. If $\lambda _n=\lambda$ and $\mu _n=\mu$ are constant, the equation is easily solved with a generating function

1.2 $$\begin{aligned} G(s,t)=\sum _0^\infty p_ns^n, \end{aligned}$$

and one finds that

1.3 $$\begin{aligned} G_t+(\lambda s-\mu )(1-s)G_s=0, \end{aligned}$$

and the solution starting with *m* individuals ($G=s^m$ at $t=0$) is

1.4 $$\begin{aligned} G=\left[ \frac{\lambda s-\mu +\mu (1-s)e^{(\lambda -\mu )t}}{\lambda s-\mu +\lambda (1-s)e^{(\lambda -\mu )t}}\right] ^m.\end{aligned}$$

The mean of the population is

1.5 $$\begin{aligned} N=\left. \frac{\partial {G}}{\partial {s}}\right| _{s=1}, \end{aligned}$$

and it is simple to show directly from (1.3) that *N* satisfies the mean field equation

1.6 $$\begin{aligned} {\dot{N}}=(\lambda -\mu )N. \end{aligned}$$

The probability of extinction, $p_0=G(0,t)$, is given by

1.7 $$\begin{aligned} p_0=\left[ \frac{\mu \left\{ e^{(\lambda -\mu )t}-1\right\} }{\lambda e^{(\lambda -\mu )t}-\mu }\right] ^m=\left[ \frac{\mu \left\{ 1-e^{-(\mu -\lambda )t}\right\} }{\mu -\lambda e^{-(\mu -\lambda )t}}\right] ^m, \end{aligned}$$

and we see that

1.8 $$\begin{aligned} p_0(\infty )=\left( \frac{\mu }{\lambda }\right) ^m,\quad \lambda >\mu ;\qquad p_0(\infty )=1,\quad \lambda <\mu . \end{aligned}$$

If the population is growing ($\lambda >\mu )$ and the initial population size is of reasonable size ($m\gg 1$), then the likelihood of extinction is negligible; on the other hand a decaying population ($\lambda <\mu$) will eventually become extinct. Essentially the same conclusion is true if $\lambda$ and $\mu$ are functions of *t*.

### Nonlinear Stochastic Models

The problem with such discrete models is that their extension to nonlinear processes becomes less tractable. As the simplest example, suppose that the birth rate $\lambda$ is constant, but that the specific death rate is $\mu _n=\dfrac{\lambda n}{K}$. In this case we would expect that the mean of the population would satisfy the Verhulst (1845) logistic equation, with carrying capacity *K*. There is a large literature dealing with such problems; see for example Bartlett et al. (1960), Nåsell (2001), Ovaskainen and Meerson (2010) and Doering et al. (2005). The paper by Ovaskainen and Meerson, in particular, contains many further references. We summarise some of the results here, though perhaps in a slightly different guise.

The equation (1.1) takes the form

$$\begin{aligned}{\dot{p}}_n=-\Delta _-(np_n)+\frac{1}{K}\Delta _+(n^2p_n),\end{aligned}$$

1.9 $$\begin{aligned} \Delta _-q_n=q_n-q_{n-1},\quad \Delta _+q_n=q_{n+1}-q_n, \end{aligned}$$

and the generating function defined in (1.2) now satisfies

1.10 $$\begin{aligned} G_\tau +s(1-s)G_s=\frac{(1-s)}{K}\left( sG_s\right) _s, \end{aligned}$$

where we have scaled time by writing $\tau =\lambda t$. As is commonly done, the subscripts $\tau$ and *s* in (1.10) denote partial derivatives (but the subscripts *n*, etc. in (1.9) are indices).

#### Generating Function

The carrying capacity *K* is an integer, and if it is not large, we would expect extinction to occur fairly rapidly. In fact, eventual extinction is certain for any finite *K*, in the sense that $G\rightarrow 1$ as $\tau \rightarrow \infty$ (thus $p_0(\infty )=1$). On the face of it, this seems to indicate that a continuum model is doomed. If we consider (1.9) for $n=0,1,\ldots$, it is not difficult to show, since $p_0$ is bounded above and thus ${\dot{p}}_0\rightarrow 0$ as $\tau \rightarrow \infty$, that $p_n\rightarrow 0$ for all $n\ge 1$. The two possibilities are that the mean of the population grows unboundedly, or that extinction occurs. The effect of the diffusion term in (1.10) appears to imply the second of these conclusions. If we write $G=1+g$ (so *g* also satisfies (1.10), but its steady state is $g=0$), then we find

1.11 $$\begin{aligned} \tfrac{1}{2}K\frac{d {}}{d {\tau }}\int _0^1\frac{e^{-Ks}g^2\,\hbox {d}s}{1-s}=-\int _0^1sg_s^2e^{-Ks}\,\hbox {d}s, \end{aligned}$$

and thus indeed $g\rightarrow 0$ (since $g=0$ at $s=1$). The rate of approach can be estimated by writing $g=\phi (s)e^{\sigma \tau }$, and then conversion of (1.10) to Sturm–Liouville form

1.12 $$\begin{aligned} \left\{ se^{-Ks}\phi _s\right\} _s=\frac{\sigma Ke^{-Ks}}{1-s}\,\phi ,\quad \phi (1)=0, \end{aligned}$$

shows that

1.13 $$\begin{aligned} -\sigma =\mathrm{inf}\,\left[ \frac{\displaystyle {\int _0^1se^{-Ks}\phi '{}^2\,\hbox {d}s}}{\displaystyle {K\int _0^1\dfrac{e^{-Ks}\phi ^2\,\hbox {d}s}{1-s}}}\right] , \end{aligned}$$

where the admissible functions $\phi$ are piecewise smooth functions with $\phi (1)=0$. A simple estimate (which also betrays the boundary layer structure of the eigenfunctions) is to take

1.14 $$\begin{aligned} \phi =\left\{ \begin{array}{ll} 1,&{}\quad s<1-\dfrac{\lambda }{K},\\ &{}\\ \dfrac{K(1-s)}{\lambda },&{}\quad s>1-\dfrac{\lambda }{K},\end{array}\right. \end{aligned}$$

whence we find for large *K* that  (1.54 is the minimum of $\dfrac{e^\lambda -1}{\lambda ^2}$).

From this we see that for large *K*, extinction takes an exponentially large time to occur. This is similar to the Ehrenfest urn problem and is not a matter for concern when *K* is large, but for *O*(1) values of *K*, extinction will occur on times $\tau \sim O(1)$. In practice, the distribution approaches a quasi-steady state (Bartlett et al. 1960), which may be determined as follows. In a steady state, (1.9) implies

1.15 $$\begin{aligned} p_n=\frac{(n-1)K}{n^2}\,p_{n-1},\quad n\ge 1, \end{aligned}$$

and obviously $p_0=1$, $p_n=0$ for $n>1$. The quasi-steady-state assumption is that $p_n$ for $n\ge 1$ approaches a (quasi-)steady state while $p_0<1$; the idea is that $p_0$ varies on a slow time scale. If this is the case then we can solve (1.15) to obtain

1.16 $$\begin{aligned} p_n=\frac{AK^n}{n\,n!},\quad n\ge 1, \end{aligned}$$

where *A* is slowly varying. Applying $G=1$ at $s=1$, this gives

1.17 $$\begin{aligned} G=p_0+\frac{(1-p_0)\displaystyle {\int _0^s\dfrac{(e^{Kx}-1)\,\hbox {d}x}{x}}}{\displaystyle {\int _0^1\dfrac{(e^{Kx}-1)\,\hbox {d}x}{x}}};\end{aligned}$$

we can then use this to calculate

1.18 $$\begin{aligned} {\dot{p}}_0=\frac{(1-p_0)}{\displaystyle {\int _0^1\dfrac{(e^{Kx}-1)\,\hbox {d}x}{x}}}\approx Ke^{-K}(1-p_0), \end{aligned}$$

using Laplace’s method, which confirms the quasi-steady-state hypothesis and is also consistent with the earlier estimate of decay rate. The next question is to provide this quasi-stationary profile. This can be obtained from (1.16) using Stirling’s approximation, but it is more illuminating to use a continuum approximation for $p_n$ in order to show that it is obtained on a timescale of $\tau \sim O(1)$.

#### Continuum Approximation

Keeping *K* large (when we therefore might expect the continuous model to apply), we revert to (1.1), and then writing

1.19 $$\begin{aligned} n= K\xi ,\quad p_n=\frac{p(\xi ,\tau )}{K},\quad \Delta \xi =\frac{1}{K}, \end{aligned}$$

(1.1) is a discrete approximation to

1.20 $$\begin{aligned} p_\tau +[\xi (1-\xi )p]_\xi =0,\quad \int _0^\infty p\,\hbox {d}\xi =1, \end{aligned}$$

which can be solved using the method of characteristics, for example with an initial condition $p=\delta (\xi -\xi _0)$, where $K\xi _0$ is the initial population size. It can then be shown that the continuous Verhulst model for the mean population is regained. A nice way to demonstrate this uses the fact that with a delta function as initial condition, the solution must in fact be

1.21 $$\begin{aligned} p=\delta [\xi -N(t)]. \end{aligned}$$

Substituting this in to (1.20), we find, using the language of generalised functions,

1.22 $$\begin{aligned} {[}-{\dot{N}}+\xi (1-\xi )]\delta '(\xi -N)+(1-2\xi )\delta (\xi -N)=0; \end{aligned}$$

now we multiply by $(\xi -N)$ and use the fact that $x\delta (x)=0$ and thus $x\delta '(x)=-\delta (x)$ to obtain $[{\dot{N}}-\xi (1-\xi )]\delta (\xi -N)=0$, whence we derive the logistic equation

1.23 $$\begin{aligned} {\dot{N}}=N(1-N) \end{aligned}$$

on integrating over $0<\xi <\infty$.

An extension to this for large *K* is to use the next term in the expansion of (1.1), which leads to the Fokker–Planck equation

1.24 $$\begin{aligned} p_\tau +\{\xi (1-\xi )p\}_\xi =\frac{1}{2K}\{\xi (1+\xi )p\}_{\xi \xi }, \end{aligned}$$

and to solve this, we write

1.25 $$\begin{aligned} \xi =N(\tau )+\frac{\eta }{\sqrt{K}},\quad p=\sqrt{K}\phi (\eta ,\tau ),\quad \int _{-\infty }^\infty \phi \,\hbox {d}\eta \approx 1, \end{aligned}$$

and if we choose *N* to satisfy (1.23), then

1.26 $$\begin{aligned} \phi _\tau +(1-2N)(\eta \phi )_\eta \approx \tfrac{1}{2}N(1+N)\phi _{\eta \eta }. \end{aligned}$$

As $\tau \rightarrow \infty$, $N\rightarrow 1$, and (1.26) has a quasi-steady solution

1.27 $$\begin{aligned} \phi =\frac{1}{\sqrt{2\pi }}\,e^{-\frac{1}{2}\eta ^2}. \end{aligned}$$

There is a caveat to this result. This is because the approximation in (1.27) is invalid for $\eta \sim \sqrt{K}$. To deal with this, we revert to *p* and $\xi$, and since the far-field expression in (1.27) is

1.28 $$\begin{aligned} p\approx \sqrt{\frac{K}{2\pi }}\,e^{-\frac{1}{2}K(\xi -1)^2}, \end{aligned}$$

we define

1.29 $$\begin{aligned} p=A(\tau )\,e^{K\psi }, \end{aligned}$$

where we assume that , so that (1.27) corresponds to $\psi \approx -\tfrac{1}{2}(\xi -1)^2$, $A=\sqrt{\dfrac{K}{2\pi }}$, for $\xi \approx 1$. We use the language of the operational calculus ($f(\xi +h)=e^{hD}f(\xi )$, where $D=\displaystyle {\frac{\partial {}}{\partial {\xi }}}$), to write (1.9) in the form

1.30 $$\begin{aligned} p_\tau =K\left[ \left\{ \exp \left( -\frac{D}{K}\right) -1\right\} (\xi p)+\left\{ \exp \left( \frac{D}{K}\right) -1\right\} (\xi ^2 p)\right] . \end{aligned}$$

Using the fact that $\displaystyle {\dfrac{D}{K}f(\xi )e^{K\psi }\approx \psi _\xi f(\xi )e^{K\psi }}$, we then find that (1.30) takes the approximate form

1.31 $$\begin{aligned} \psi _\tau =Q=\xi (e^{-P}-1)+\xi ^2(e^P-1),\quad P=\psi _\xi . \end{aligned}$$

The initial condition we choose for (1.31) should correspond approximately to an initial delta function, which we take to be centred at the steady value $\xi =1$. Note that an arbitrary additive constant for $\psi$ can be absorbed into *A*. To represent the initial condition, we will consider the family of functions

1.32 $$\begin{aligned} \psi =-\tfrac{1}{2}a(\xi -1)^2\ \ \mathrm{at}\ \ \tau =0, \end{aligned}$$

where the limit of $a\rightarrow \infty$ corresponds to a delta function.

The equation (1.31) is a nonlinear hyperbolic equation of the form

1.33 $$\begin{aligned} F(\xi ,P,Q)=Q-\xi (e^{-P}-1)-\xi ^2(e^P-1)=0, \end{aligned}$$

which can be solved by writing it in characteristic form using Charpit’s equations. The initial condition (1.32) can be written in parametric form as

$$\begin{aligned} \xi =\sigma ,\quad \psi =\psi _0(\sigma )=-\tfrac{1}{2}a(\sigma -1)^2,\quad P=P_0(\sigma )=-a(\sigma -1), \end{aligned}$$

1.34 $$\begin{aligned} Q=Q_0(\sigma )=\sigma [e^{a(\sigma -1)}-1]+\sigma ^2[e^{-a(\sigma -1)}-1]\ \ \mathrm{at}\ \ \tau =0, \end{aligned}$$

and Charpit’s equations reduce to

1.35 $$\begin{aligned} Q= & {} Q_0(\sigma ),\dfrac{}{}\nonumber \\ P= & {} \ln \left\{ \frac{1}{2\xi ^2}\left[ (\xi ^2+\xi +Q){\pm }\left\{ (\xi ^2+\xi +Q)^2-4\xi ^3\right\} ^{1/2}\right] \right\} \equiv P(\xi ,\sigma ),\dfrac{}{}\nonumber \\ {\dot{\xi }}= & {} \xi e^{-P}-\xi ^2 e^P={\mp } \left\{ (\xi ^2+\xi +Q)^2-4\xi ^3\right\} ^{1/2},\dfrac{}{}\nonumber \\ {\dot{\psi }}= & {} Q+P{\dot{\xi }},\quad \Rightarrow \psi =\psi _0(\sigma )+Q\tau +\int _\sigma ^\xi P(\eta ,\sigma )\,\hbox {d}\eta , \end{aligned}$$

where the overdots refer to differentiation with respect to $\tau$. The expression for *P* comes from solving the quadratic equation for $e^P$ given by $F=0$. The upper or lower signs in (1.35) are chosen so that

1.36 $$\begin{aligned} {\dot{\xi }}_0(\sigma )=\left. {\dot{\xi }}\right| _{\tau =0}=\sigma e^{a(\sigma -1)}-\sigma ^2 e^{-a(\sigma -1)}. \end{aligned}$$

**Fig. 1:**
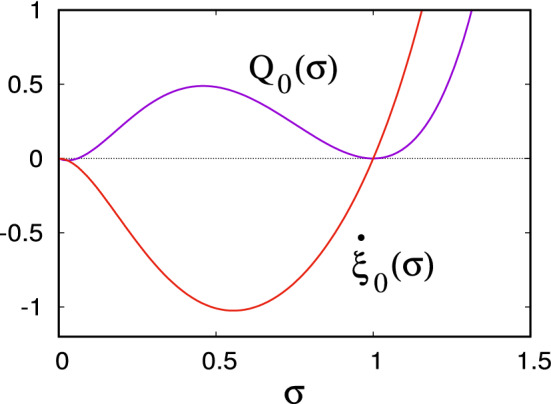
The functions ${\dot{\xi }}_0(\sigma )$ defined by (1.36) and $Q_0(\sigma )$ defined in (1.34), using a value $a=3$

This function is plotted in Fig. 1, along with $Q_0(\sigma )$. Evidently the upper sign is chosen for  and the lower one for $\xi >1$. Thus for small $\tau$, the characteristics move to the left for $\xi <1$ and to the right for $\xi >1$. This will remain true unless ${\dot{\xi }}=0$, which occurs when $Q=Q_{\pm }(\xi )$, where

1.37 $$\begin{aligned} Q_{\pm }(\xi )=-\xi (1{\pm }\xi ^{1/2})^2 \end{aligned}$$

are the roots of ${\dot{\xi }}=0$. As suggested by Fig. 1, this does not occur, since $Q_0(\sigma )>0$ (except near $\sigma =0$, discussed below).

**Fig. 2:**
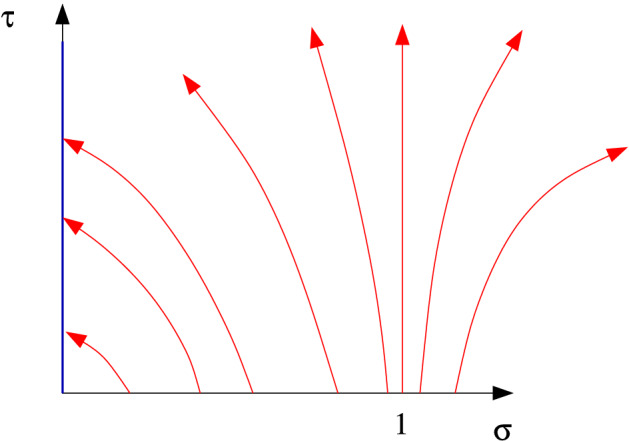
Schematic characteristic diagram for (1.35)

The form of the characteristic diagram is then shown in Fig. 2. For $\xi <1$, ${\dot{\xi }}<0$ and for $\xi >1$, ${\dot{\xi }}>0$. Therefore at large $\tau$, all the characteristics come from the vicinity of $\sigma =1$, where $Q\approx 0$, and so from (1.35),

1.38 $$\begin{aligned} {\dot{\xi }}\approx \xi ^2-\xi ,\quad P\approx -\ln \xi ,\quad \psi \approx \xi \left( \ln \frac{1}{\xi }+1\right) -1, \end{aligned}$$

which provides the uniformly valid quasi-steady solution for $\psi$; note that $\psi \sim -\tfrac{1}{2}(\xi -1)^2$ as $\xi \rightarrow 1$.

A comment is necessary concerning the behaviour at $\xi =0$. With the precise choice in (1.32), it is clear from (1.34) that for any finite value of *a*, $Q_0<0$ for sufficiently small $\sigma$, and also ${\dot{\xi }}>0$ there. Thus for large but finite *a*, a shock will form near $\xi =0$. This would slightly confuse matters, but in fact this issue is associated with the consequence at finite *a* that the probability density $p>0$ at $\xi =0$. In reality, a better initial condition would have $\psi \rightarrow -\infty$ at $\sigma =0$, so that this region of ${\dot{\xi }}>0$ disappears, but the limit is a non-uniform one, since $\xi =0$ remains a characteristic. It seems to be that the resultant shock is the cause of the necessity of treating $p_0$ separately.

#### Long-Time Evolution

The key to extending the result above is to realise that the correct way to formulate a ‘continuous’ distribution model is to allow *p* to have delta function behaviour at $\xi =0$. In keeping with what the discrete model actually implies, we adopt (1.29) and thus (1.30) for $\xi$ strictly positive, and let the probability $p_0(\tau )$ at $\xi =0$ be finite. Thus the distribution is a Stieltjes one, and we have

1.39 $$\begin{aligned} p_0+\int _{0+}^\infty p(\xi ,\tau )\,\hbox {d}\xi =1, \end{aligned}$$

where the lower limit $0+$ indicates that it is in fact slightly positive ($=\dfrac{1}{K}$). Using (1.38) (which shows that $\psi \approx -\tfrac{1}{2}(\xi -1)^2$ near $\xi =1$) together with the use of Laplace’s method for the integral, we find

1.40 $$\begin{aligned} A\approx \sqrt{\frac{K}{2\pi }}(1-p_0), \end{aligned}$$

where *A* is as in (1.29).

The approximation in (1.31) is essentially that used in the geometric optics approximation of WKB theory (Bender and Orszag 1978), but to obtain a result equivalent to (1.18), we need the next term of the approximation. To find this, we return to (1.30), but now written in the form

1.41 $$\begin{aligned} \psi _\tau =\xi _- e^{K\psi (\xi _-)}-\xi e^{K\psi }+\xi _+^2e^{K\psi (\xi _+)}-\xi ^2 e^{K\psi },\quad \xi _{\pm }=\xi {\pm }\frac{1}{K}, \end{aligned}$$

and we write $\psi =\Psi _0+\dfrac{1}{K}\Psi _1+\ldots$; at leading order we regain (1.31); selecting the steady solution $\Psi _0=\xi \left( \ln \dfrac{1}{\xi }+1\right) -1$, the equation for $\Psi _1$ is easily solved to find $\Psi _1=-\frac{3}{2}\ln \xi$, so that the physical optics approximation for *p* is

1.42 $$\begin{aligned} p\approx A\xi ^{-3/2}\exp \left[ K\left\{ \xi \left( \ln \frac{1}{\xi }+1\right) -1\right\} \right] . \end{aligned}$$

This of course looks suspicious at low $\xi$, but it seems to be all right provided we take $\xi \ge \dfrac{1}{K}$. In fact, we now derive the equation for $p_0$ by taking

1.43 $$\begin{aligned} {\dot{p}}_0=\frac{p_1}{K}=\frac{1}{K^2}\,p\left( \frac{1}{K},\tau \right) \approx \frac{e}{\sqrt{2\pi }}\,Ke^{-K}\,(1-p_0). \end{aligned}$$

This can be favourably compared to (1.18), since $\dfrac{e}{\sqrt{2\pi }}\approx 1.084$. Actually, we can see what is happening here, since $\dfrac{e}{\sqrt{2\pi }}$ is just Stirling’s (rather good) approximation to $\dfrac{1}{n!}$ in (1.16) when $n=1$. Thus the continuous probability density function approximation to $p_n$ does rather well all the way down to $n=1$.

The implication of this discussion is that when a continuous model indicates a very small population being maintained for a significant time, then in practice the population will become extinct. The importance of this lies in the fact that it is not uncommon for population models which exhibit oscillations to have precisely this property, and suggests that where the oscillations are the point of focus, the continuous models are in essence incorrect.

A second conundrum which relates continuous models to low population densities is what I will call the frogspawn problem. Frogs produce thousands of eggs (e. g., Beattie 1987), but only a few of these survive to become adult frogs (Calef 1973). If we want to write a continuous model for a frog population which describes the production of thousands of eggs and their maturation as tadpoles to adult frogs, we need to find a way in which a small number of the original eggs can survive. There need to be very few, but importantly these few must not go extinct; how can that be? In a later section, I will describe one possible answer to this query.

## Atto-Foxes

An enduring issue in population dynamics is what has been called ‘the atto-fox problem’ (Lobry and Sari 2015). The origin of this term lies in a model suggested by Anderson et al. (1981) to account for the fact that rabies outbreaks tend to recur, and they explained this by showing that oscillations can occur in the model. The work was extended by Murray et al. (1986) to account both for the recurrence and also the spread of the disease by including a term allowing for the ‘diffusion’ of wandering rabid foxes. An account of the model is given in his book (Murray 1989, chapter 20), since re-published in second and third editions. The epidemiology of fox rabies is described by Bacon (1985) and Toma and Andral (1977) for example. The virus is expressed in the saliva and transmitted by biting. Models of rabies dynamics continue to attract attention (e. g., Liu et al. 2017). However, the simple early models of Anderson and May and of Murray suffer from a defect, which is that the minima of the infected population reach levels which are so low as to imply extinction of the virus. Mollison (1991, p. 31) severely criticised (‘this is incredible’) the continuous model on two counts, the second of which is the inability of a continuous model to allow populations to become extinct, despite reaching values (in the rabies case) of one atto-fox ($10^{-18}$ of a fox) per square kilometre.

Mollison’s advice was that it is essential to use a stochastic model instead of a continuous one, with the consequence of extinction. One way in which local extinction can be circumvented is through spatial heterogeneity. Most simply, if a population is distributed between relatively isolated regions with some contact, then its eradication in one region can be overcome by leakage from a neighbouring patch. For example, measles in the UK in pre-vaccination times (before 1965) was endemic in London but was transmitted to smaller communities (below the critical size necessary to maintain the endemic state) by spatial transmission (Grenfell et al. 2001; Korevaar et al. 2020), in much the same way as contraction of the heart muscle is enabled by spread of the pacemaker activity of the sino-atrial node cells to the excitable cardiac tissue cells.

The simplest way to think about spatial transmission is by the addition of a diffusion term. Of course, this only describes nearest neighbour contacts and is not suitable to describe local outbreaks caused by long-range transmission of ‘sparks’ (Grenfell et al. 2001), for which a spatial convolution integral would be more appropriate, but it will serve for the present discussion. It is well known that the addition of diffusion to an oscillatory system leads to periodic travelling waves. However, if the minima of the oscillations are so low as to promote extinction, then, as for measles or the heart, a local outbreak will cause the propagation of a solitary travelling wave

Here I want to explore a different possibility, which is that there is something fundamentally lacking in such models, and that is the concept of a reservoir. Let me illustrate this by reference to a simple (dimensionless) population model

2.1 $$\begin{aligned} \varepsilon {\dot{f}}=sf-f, \end{aligned}$$

where $\varepsilon \ll 1$ represents the idea that the timescales for growth and decay of the population *f* are much smaller than that of the slowly varying nutrient source *s*. We shall see examples built around (2.1) in the following section. If $s<1$, then the population plummets and will become extinct in practice. A reservoir allows for a second state *g* and the transfer $f\rightarrow g$ is enabled at low values of *s*; basically, the population hibernates until *s* increases above one, at which point the awakening $g\rightarrow f$ is enabled. This is somewhat similar to the concept of a refuge (Sih 1987), but it is not quite the same.

In the case of rabies, I suggested (Fowler 2000) that a resolution of the issue could be found in the distribution of infection times. SIR-type models (with a rate of recovery of the infected population *I* of *rI*) are equivalent to assuming an exponential distribution of recovery times $R(a)=1-e^{-ra}$, where *a* is the ‘age’ of the infection, so that $R'(a)=re^{-ra}$ is the recovery time probability density (Fowler and Hollingsworth 2015). But such an assumption is not commonly realistic; if one instead assumes a fixed disease period, then the SIR model reduces to a differential-delay equation (Soper 1929). A more general assumption is that of a gamma distribution (Fowler and Hollingsworth 2015) of the form

2.2 $$\begin{aligned} R'(a)=\frac{r^\gamma a^{\gamma -1}e^{-ra}}{\Gamma (\gamma )} , \end{aligned}$$

which provides a gradual change from the exponential to fixed period distributions as $\gamma$ increases from 1 to $\infty$. In fact, experimental work in the 1960s (Parker and Wilsnack 1966; Steck and Wandeler 1980) indicated that though most rabid foxes would have incubation periods of about a month, around 10% of cases would survive for up to six months; in effect these more resistant foxes act as a reservoir for the virus. The point is that it is the small ratio of incubation time to population growth time which causes the exponentially small minima to occur.

**Fig. 3:**
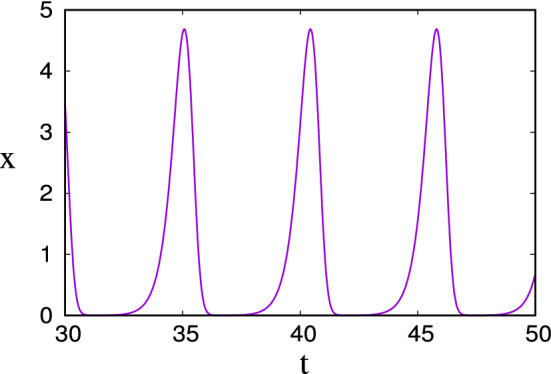
Solution of the delayed logistic equation (3.1) at $\alpha =2.5$. The asymptotic limit of large $\alpha$ is indicated by the flat minimum phase (the minimum of *x* is approximately 0.00158)

## Boom-and-Bust Dynamics

Oscillations in continuous population models which have extremely low minima occur in many other situations. One simple but remarkable example is the delayed logistic equation (Hutchinson 1948), which can be written in the form

3.1 $$\begin{aligned} {\dot{x}}=\alpha x(1-x_1),\quad x_1\equiv x(t-1). \end{aligned}$$

In this dimensionless form, $\alpha$ is the ratio of the delay to the specific growth rate, and large values cause periodic solutions to occur with extremely small minima. What is remarkable is that oscillations only occur for $\alpha >1.57$, but already for $\alpha =2.5$ the asymptotic limit is visible. This is shown in Fig. 3. The minimum of the oscillations is approximately

3.2 $$\begin{aligned} x_{\min }\approx \alpha \exp (-e^\alpha +2\alpha -1) \end{aligned}$$

(Fowler 1982) and is already of order $10^{-3}$ when $\alpha =2.5$.

While the model itself is now largely only of academic interest, it is closely related to a simple model of the immune response to an infecting antigen presented by Dibrov et al. (1977a, 1977b), which was analysed by Fowler (1981). In its simplest dimensionless form, the model is given by

3.3 $$\begin{aligned} {\dot{g}}= & {} \alpha g(1-a),\dfrac{}{}\nonumber \\ {\dot{a}}= & {} \beta [g_1-a\{1-\kappa +\kappa g\}],\quad g_1=g(t-1), \end{aligned}$$

where *a* and *g* denote antibody and antigen densities, respectively. The interpretation of the model is fairly straightforward: the infecting antigen grows but is removed by the antibodies, produced here through stimulation of the humoral immune response, the maturation time of which produces the delay in the response. If

3.4 $$\begin{aligned} \kappa >\kappa _c=\alpha e^{1-\alpha }, \end{aligned}$$

then the antigen grows unboundedly, otherwise oscillations occur, and these are severe if the delay is large. Specifically, if $\alpha$ and $\beta$ are large (and thus necessarily $\kappa$ is small), then we have $a\approx g_1$, and we regain the logistic delay equation. The behaviour of the variables is similar to that shown in Fig. 3.

The immune response time is of the order of days, and much larger than a common infective (e. g., viral) growth time, and it is because of this in the model that the minima in the oscillations are attometric in scale. The immune system is a good deal more complicated than indicated in (3.3), but can be modelled in similar fashion, usually with a continuous model (Perelson 2002), and sometimes with delays (Lee et al. 2009; Rundell et al. 1998) or without (Yan et al. 2016). Commonly extinction occurs, in the sense that viral populations become very low, with the assumption that stochastic elimination occurs at these levels (Yan et al. 2016), but this is not always the case: HIV is one viral disease where an endemic state is maintained for a long period (Perelson 2002), and the herpes virus establishes itself in the body by entering a dormant state which allows for further outbreaks (Nicoll et al. 2012).

### Microbial Growth

Microbial growth models are another source of oscillatory dynamics. A particularly simple example is a model due to Omta et al. (2013), who were interested in oscillations in oceanic calcifiers as a possible cause of periodic ice ages. Their model can be written as

3.5 $$\begin{aligned} {\dot{B}}= & {} kYCB-dB,\dfrac{}{}\nonumber \\ {\dot{C}}= & {} I-kCB; \end{aligned}$$

here *B* represents biomass, and *C* a (limiting) nutrient (in Omta *et al*. ’s case the nutrient is carbon and the biomass consists of calcifiers such as coccolithophores). Very similar types of model have been used in describing oscillations in glycolysis (Goldbeter 1996), and plankton blooms (Huppert et al. 2005; Mahadevan et al. 2012). *I* represents a rate of input of nutrient to the system. It can be noted that if $I=0$, (3.5) is just an SIR model, in which nutrient represents susceptibles, and the biomass masquerades as the infected. In the present case, the non-zero supply allows recovery, much as the increasing fox population allows oscillation in the rabies model.

By defining the non-dimensional variables and parameter

3.6 $$\begin{aligned} B=\frac{IY}{d}b,\quad C=\frac{d}{kY}c,\quad t\sim \frac{1}{\sqrt{IkY}},\quad \varepsilon =\frac{\sqrt{IkY}}{d}, \end{aligned}$$

the model can be written in the dimensionless form (cf. Fowler 2013)

3.7 $$\begin{aligned} \varepsilon {\dot{b}}= & {} (c-1)b,\dfrac{}{}\nonumber \\ {\dot{c}}= & {} \varepsilon (1-bc), \end{aligned}$$

where the small parameter $\varepsilon$ is a measure of the ratio of the nutrient supply rate to the biomass growth rate, and the nature of the model is easily understood by writing $b=e^\theta$, whence we have

3.8 $$\begin{aligned} \ddot{\theta }+V'(\theta )=-\varepsilon e^\theta {\dot{\theta }}, \end{aligned}$$

where the potential *V* is defined by

3.9 $$\begin{aligned} V(\theta )=e^\theta -\theta . \end{aligned}$$

**Fig. 4:**
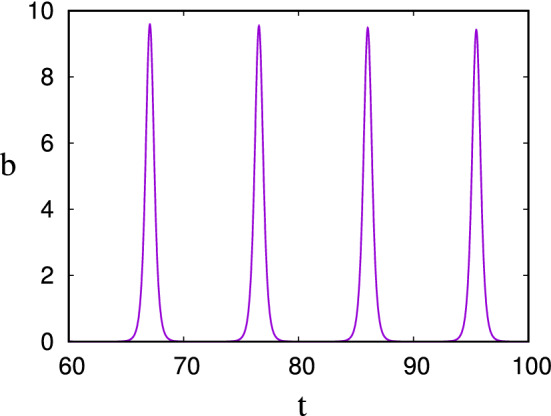
The solution for *b* in (3.7) using a value of $\varepsilon =0.001$ and initial conditions $b=10$, $c=1$. The large initial value of *b* causes the minima to be exponentially small; here the minimum at $t\approx 90.75$ is $b\approx 0.00074$, for example

When $\varepsilon \ll 1$, this is a slowly decaying nonlinear oscillator, and if the ‘energy’ $E=\tfrac{1}{2}{\dot{\theta }}^2+V(\theta )$ is large, then the biomass *b* exhibits typically spiky ‘boom-and-bust’ oscillations. The form of these is shown in Fig. 4.

**Fig. 5:**
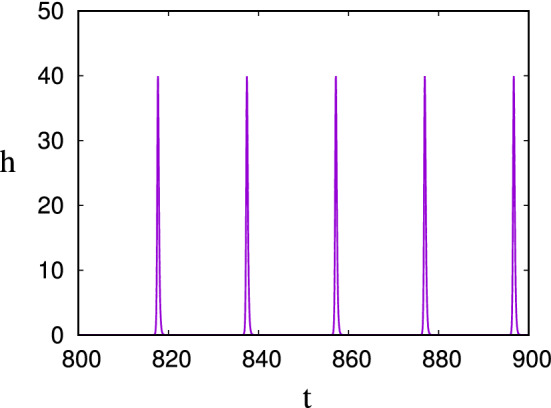
The solution for *h* in (3.10) using values $\varepsilon =0.1$ and $\delta =0.5$. The minimum of the population is $\sim 10^{-15}$

Fowler (2014) extended this model to describe competing microbial populations (heterotrophs and fermenters). Denoting the heterotroph and fermenter populations as *h* and *f*, his model is, in dimensionless form,

3.10 $$\begin{aligned} \varepsilon {\dot{h}}= & {} \delta hs+hc-h,\dfrac{}{}\nonumber \\ \varepsilon {\dot{f}}= & {} sf-f,\dfrac{}{}\nonumber \\ {\dot{s}}= & {} \varepsilon (1-sf-\delta hs),\dfrac{}{}\nonumber \\ {\dot{c}}= & {} \varepsilon (sf-hc); \end{aligned}$$

here *s* and *c* are two different forms of organic carbon. The heterotrophs can utilise both, whereas the fermenters can only use the *s*-form, but produce the *c*-form, which is preferentially used by the heterotrophs. The system thus has the form of an activator-inhibitor system, and, as shown in Fig. 5, boom-and-bust oscillations occur in conditions of starvation i. e., for small $\varepsilon$. For $\varepsilon =0.2$, the minimum of *h* is $\approx 1.9\times 10^{-4}$, but for $\varepsilon =0.1$, as shown in the figure, it is $\approx 1.8\times 10^{-15}$. When it is reduced slightly further to the practically estimated value of $\varepsilon =0.07$ (Fowler et al. 2014), the microbial dimensionless minima are of the order of $10^{-32}$, and extinction beckons. Given a common bacterial loading of $10^9$ cells g$^{{-1}}$ (Kirchman 2012, p. 9), this last value corresponds to levels of 1 yocto-cell per gram, thus transcending Mollison’s atto-fox per km$^{{2}}$.

In all these examples, extinction looms, and the oscillations are suspect. I want to suggest that extinction can be avoided in reality by the presence of a reservoir. This concept resembles but is distinct from that of a refuge for prey in predator–prey models (e. g., Sih 1987; Haque et al. 2014; Balaban-Feld et al. 2019), although the introduction of that concept was aimed at providing stability for the otherwise structurally unstable Lotka-Volterra model. The purpose and function of a reservoir in the present case is quite different. In the case of the rabies virus, a reservoir could take the form of an endemic host, which could even be the long-lived foxes themselves. But, as pointed out by Sterner and Smith (2006), there are actually many different reservoirs amongst mammals for the rabies virus and its variants. From the point of view of a continuous model, all that is necessary is that there is at least one reservoir where viral extinction does not occur.

In the case of microbes, the existence of bacteria in a dormant state is well recognised (Kaprelyants et al. 1993; Lennon and Jones 2011; Hoehler and Jørgensen 2013). Fowler and Winstanley (2018) suggested a simple model to describe the switch to dormancy; their model can be written in dimensionless form as

3.11 $$\begin{aligned} \varepsilon {\dot{b}}= & {} (c-1)b -qb+ pa,\dfrac{}{}\nonumber \\ {\dot{c}}= & {} \varepsilon (F-bc),\dfrac{}{}\nonumber \\ \varepsilon {\dot{a}}= & {} qb-pa, \end{aligned}$$

which generalises (3.7). Here *a* represents the dormant state, and *p* and *q* are switching functions which switch on (*q*) and off (*p*) at low nutrient (*c*) or active biomass (*b*) levels. This model allows self-sustained oscillations when $\varepsilon$ is small, again of boom-and-bust type, and although generally they have less extreme minima than the yocto-cell example, effective extinction of the active biomass can occur; the difference is that the dormant bacterial population remains viable when that happens, providing a nursery for bacterial growth when the environmental stress is reduced. *The reduction of b to tiny levels does not matter.* This suggestion of a latent reservoir is one that can come to the rescue of continuous models when they indicate extinction. One circumstance where a reservoir may not exist is in a viral infection. Many viral infections may be completely eliminated (Rundell et al. 1998; Yan et al. 2016), although there are some where an endemic or latent state is established (Perelson 2002; Nicoll et al. 2012).

## Frogspawn

Finally I want to consider another problem of small numbers and whether it can be catered for in a continuous model. Many species of plants and animals reproduce by means of the production of thousands, or even millions, of eggs or seeds. Fish provide one example (e. g., Pope et al. 2010), and trees another (Greene and Johnson 1994); helminths, discussed in the conclusions, provide another. In such cases, very few of the offspring survive to adulthood, and the question is: why? I will refer to this as the frogspawn problem, as frogs provide another example of such extreme fecundity.

The population biology of frogs (it should be noted that there are many different species) has been studied by many authors (Berven 1990; Friedl and Klump 1997; Heyer et al. 1975; Smith 1983; Travis et al. 1985). Frogs produce thousands of eggs (Gibbons and McCarthy 1986), some of which later hatch to tadpoles, and still fewer of these make it to adulthood. Roughly speaking, a single adult frog in a stable population ought to produce a single offspring. How can this be?

The reason I find this perplexing is that the normal predation rate of a population *F* would be $\propto F$ (assuming plenty of predators) so that if the population becomes very low, we arrive at the previous conundrum: why does extinction not occur?

There is in fact a simple possible answer to this problem. Let us consider a population of adult frogs *F*, and suppose that $F_n$ is the frog population measured at intervals of $\Delta T=1$ year, thus $F_n=F(n\Delta T)$. If each female produces *N* eggs per year ($N\sim 1000$ year$^{{-1}}$), and the adult death rate is $\mu$ (year$^{{-1}}$), then the year on year change in the population would be

4.1 $$\begin{aligned} F_{n+1}-F_n=\Delta F=M_n\Delta T-\mu F_n\Delta T, \end{aligned}$$

where $M_n$ (frog year$^{{-1}}$) is the total number of eggs which survive predation and metamorphose to adults. In the absence of predation, $M_n=\tfrac{1}{2}NF_n$.

The death rate is enhanced for eggs and tadpoles by predation (Waller 1973)); the reason so few tadpoles survive to adulthood is that they are consumed (even by themselves). So the principal loss term is not natural death but juvenile predation. And here is the idea: when the population levels become low, the predators do not find the prey so easily. We can for example model egg and tadpole predation during the year as a term $-kM^2$ , where $M=\tfrac{1}{2}NF_n$ initially, and $\displaystyle {\frac{d {M}}{d {t}}=-kM^2}$, whence we find

4.2 $$\begin{aligned} M_n=\frac{\tfrac{1}{2}NF_n}{1+\tfrac{1}{2}kNF_n\Delta T}. \end{aligned}$$

There is in fact experimental evidence that this description may be reasonably accurate (see Brockelman 1969, figure 4). A suggestive continuous version of (4.1) is

4.3 $$\begin{aligned} {\dot{F}}={ \mu F\left[ \frac{F_0}{\delta F_0+F}-1\right] }, \end{aligned}$$

where the overdot denotes differentiation with respect to time, and

4.4 $$\begin{aligned} F_0=\frac{1}{\mu k\Delta T},\quad \delta =\frac{2\mu }{N}. \end{aligned}$$

A typical value of $\mu$ is 1 year$^{{-1}}$ (Berven 1990). With $N\sim 10^3$ year$^{{-1}}$, evidently $\delta \ll 1$ (this is the frogspawn problem), but infant predation leads to a stable population $F\approx F_0$, whose size is actually nothing to do with the egg production rate.

This of course is hardly a new idea and is the basis for Holling’s type 3 predator response to prey density (Holling 1959, 1961, 1973), in which the individual predator’s consumption rate as a function of prey density is S-shaped, and (for example) quadratic at low prey densities. This same idea was used in the spruce budworm model of Ludwig et al. (1978), where the birds’ predation of budworm larvae is described thus by Murray (1989, p. 5): ‘For small population densities ..., the birds tend to seek food elsewhere...’. The motivation leading to (4.3) has the same effect as the saturating term in the logistic or Verhulst equation:

4.5 $$\begin{aligned} {\dot{F}}={ rF\left( 1-\frac{F}{F_0}\right) }, \end{aligned}$$

whose right-hand side has the same unimodal shape as (4.3).

It should be noted that Verhulst’s (1845) suggestion of the logistic equation^1^ was not based on any process description, but on a wish to describe the weakening (*affaiblement*) of reproduction in the presence of limited resources. The present suggestion of a saturational model is based on quite different considerations.

The resolution of the frogspawn conundrum is simply due to the nonlinear egg predation rate, which allows an equilibrium to occur no matter how many eggs are produced, and whose size depends on the predation coefficient *k*. The surprising thing is that one normally teaches the Verhulst equation as a response to limited resources: the specific fecundity rate decreases with population size, but here the same effect is due to a quite different mechanism.

### An Age-Structured Model

Of course, (4.3), while suggestive, is rather crude. A more subtle approach is to consider an age-structured model in which the amphibian density *f*(*t*, *a*) depends on both time *t* and age *a*. For small *a*, *f* represents eggs, then tadpoles, and for $t>T$, say, adult frogs. The total amphibian density is then

4.6 $$\begin{aligned} F=\int _0^\infty f\,\hbox {d}a. \end{aligned}$$

The units of *f* are taken to be Am psf$^{{-1}}$ year$^{{-1}}$, where Am means amphibians, y is years, and psf is pond square foot. This last unit of area is by analogy with the Jones site model for spruce budworm outbreaks, where the larval density was measured as individuals tsf$^{{-1}}$, where tsf means ten square foot of susceptible branch surface area (Jones 1979; Hassell et al. 1999).

We pose the following model for *f*, in which the subscripts denote partial derivatives:

4.7 $$\begin{aligned} f_t+f_a=-r(a)f^2, \end{aligned}$$

by analogy with (4.3). The boundary and initial conditions are taken to be

4.8 $$\begin{aligned} f(0,a)=f_i(a),\quad f(t,0)=f_0(t), \end{aligned}$$

where the renewal equation for $f_0$ is taken to be

4.9 $$\begin{aligned} f_0(t)={ N}\int _T^\infty f(t,a)\,\hbox {d}a, \end{aligned}$$

indicating that mature adult frogs lay *N* eggs per year. The time *T* is the age of sexual maturity, commonly 1–2 years (e. g., Friedl and Klump 1997; Berven 1990). The units of *r* are Am$^{{-1}}$ psf, and *N* has units Am Am$^{{-1}}$ y$^{{-1}}$ $=$ y$^{{-1}}$, or eggs per frog per year.

If we define

4.10 $$\begin{aligned} R(a)=\int _0^ar(a')\,\hbox {d}a', \end{aligned}$$

then the solution is

4.11 $$\begin{aligned} f=\left\{ \begin{array}{ll} \dfrac{f_i(a-t)}{1+\{R(a)-R(a-t)\}f_i(a-t)},&{}\quad t<a,\\ &{}\\ \dfrac{f_0(t-a)}{1+R(a)f_0(t-a)},&{}\quad t>a, \end{array}\right. \end{aligned}$$

and for $t>T$ the renewal equation gives the integral delay equation for $f_0$:

4.12 $$\begin{aligned} f_0(t)={ N}\left[ \int _0^{t-T}\dfrac{f_0(s)\,\hbox {d}s}{1+R(t-s)f_0(s)} +\int _0^\infty \dfrac{f_i(\xi )\,\hbox {d}\xi }{1+\{R(t+\xi )-R(\xi )\}f_i(\xi )}\right] .\nonumber \\ \end{aligned}$$

Let us consider the form of *R*(*a*). The predation rate *r* is a rapidly decreasing function of *a* for $a<T$, so that *R* monotonically increases to a plateau at $R(T)={\bar{R}}$, say. Thereafter *R* will increase slowly, and if we suppose all frogs die of senescence by age *A*, say, then $R\rightarrow \infty$ as $a\rightarrow A$. In this case the second term in the square bracket is zero for $t>A$.

A natural scale for *R* is thus ${\bar{R}}$, and it is now convenient to scale the variables as

4.13 $$\begin{aligned} R\sim {\bar{R}},\quad t,a\sim T,\quad f=\frac{\Phi }{{\bar{R}}},\quad f_0=\frac{\phi }{{\bar{R}}},\quad A=T\alpha , \end{aligned}$$

and this leads to the equation for $\phi$,

4.14 $$\begin{aligned} \phi (t)={ \Lambda }\int _0^{t-1}\frac{\phi (s)\,\hbox {d}s}{1+R(t-s)\phi (s)},\quad t>\alpha , \end{aligned}$$

where

4.15 $$\begin{aligned} \Lambda =NT\gg 1, \end{aligned}$$

and the age structure is given by

4.16 $$\begin{aligned} \Phi (t,a)=\frac{\phi (t-a)}{1+R(a)\phi (t-a)}. \end{aligned}$$

Because of the large value of ${ \Lambda }$, it is relatively easy to solve (4.14). We begin with an example, and consider first the steady state. *R* is an increasing function, with $R(1)=1$ and $R\rightarrow \infty$ as $t\rightarrow \alpha$. As an illustration, suppose

4.17 $$\begin{aligned} R=\frac{\alpha -1}{\alpha -a},\quad a>1; \end{aligned}$$

the steady solution of (4.14) is then given uniquely by

4.18 $$\begin{aligned} 1={ \Lambda }(\alpha -1)\left[ 1+\phi \ln \left( \frac{\phi }{1+\phi }\right) \right] , \end{aligned}$$

and for large ${ \Lambda }$ this is approximately

4.19 $$\begin{aligned} \phi \approx \tfrac{1}{2}{ \Lambda }(\alpha -1). \end{aligned}$$

**Fig. 6:**
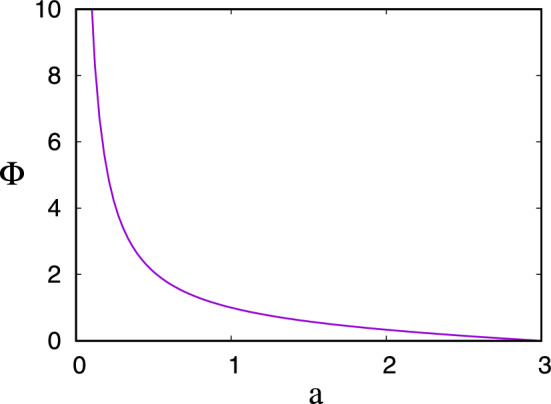
The dimensionless age distribution $\Phi$ given by (4.21), with ${ \Lambda }=2000$ and $R(a)=\dfrac{\alpha (\alpha -1)a}{\alpha (\alpha -1)-a(a-1)}$, $\alpha ={ 3}$ (corresponding to $N=1000$ y$^{{-1}}$, $T=2$ y, $A=6$ y). These are typical values for Irish frogs (Gibbons and McCarthy 1984). For visibility the range of $\Phi$ is not shown, but at $a=0$, $\Phi \approx { 1530}$

More generally, and even if time-dependent, the observation that $\phi \sim { \Lambda }$ leads to the approximate result

4.20 $$\begin{aligned} \phi \approx { \Lambda }\int _1^\alpha \frac{da}{R(a)}, \end{aligned}$$

and a uniform approximation for the age distribution is

4.21 $$\begin{aligned} \Phi =\frac{\displaystyle {\int _1^\alpha \dfrac{da}{R(a)}}}{\dfrac{1}{{ \Lambda }} +R(a)\displaystyle {\int _1^\alpha \dfrac{da}{R(a)}}}, \end{aligned}$$

which shows that the distribution descends sharply from $O({ \Lambda })$ when $a\ll 1$ to *O*(1) when $a\sim 1$. An illustration of the resulting dimensionless age distribution is shown in Fig. 6.

## Conclusions

As regards atto-foxes and yocto-cells, we suggest that the resolution of this long-standing issue may be that in practice, vanishing populations seek refuge in a safe haven, whether it be in dormancy or as an endemic remnant in another host reservoir. One example is the ability of bacteria to remain viable in the most inhospitable places (for example deep in the Earth) for an extremely long time (Hoehler and Jørgensen 2013). Plant seeds are another example: metabolic processes essentially shut down until they are stimulated to re-emerge (Bewley 1997; FitzGerald and Keener 2021).

The suggested resolution of the frogspawn problem, whether it be the logistic-type equation (4.3) or the mildly more interesting age-dependent model, seems straightforward, but it raises another issue. If the reduction of the large numbers of eggs is due to an effectively quadratic predation rate, what then is the point of the large number? We can see from (4.21) that it does not really matter how large *N* and thus $\Lambda$ is. It is possible that this is controlled by actual space limitation, but also, production of a small number of eggs presumably requires parental care, which is not so easily available for the predator-prone frog, and the large number simply indicates this (Davis and Roberts 2005).

There is a related issue in continuous modelling which arises in a classical model for human infection of the roundworm *Ascaris lumbricoides*. Roundworm infection is endemic in low-income populations with poor sanitation in tropical countries and is one of a number of ‘neglected tropical diseases’ which are a focus of much international interest (Holland 2013; Hollingsworth et al. 2015). The classic model to describe the infection was put forward by Anderson and May (1985, 1991) and takes the essential form

5.1 $$\begin{aligned} {\dot{L}}= & {} rM-\mu _2L,\dfrac{}{}\nonumber \\ {\dot{M}}= & {} \nu _0L-\mu _1M. \end{aligned}$$

Here *M* is the adult worm burden in the human small intestine, typically of the order of 10–20 per human, while *L* is the number of mature eggs in the environment. The lifetimes of eggs and adults are respectively taken to be $\mu _2^{-1}\sim$ 28–84 days, and $\mu _1^{-1}\sim 1$–2 years. Actually there is an issue here already, because viable *Ascaris* eggs in latrines have been found with ages of the order of up to 15 years (WIN-SA 2011, Chris Buckley, private communication). Leaving that aside, (5.1) is of course linear, but nonlinearity is introduced by a unimodal dependence of the recruitment rate $\nu _0$ on *M*: at low *M* this is due to the increasing probability of having male and female worms present in the human, and at high *M* because of the reduction in fecundity due to crowding. This nonlinearity allows for a stable endemic population.

Aside from the issue of the egg lifetime, there is an issue concerning the recruitment rate $\nu _0$. Anderson and May (1991) effectively avoided estimating this by using measured values of the basic reproduction rate

5.2 $$\begin{aligned} R_0=\frac{\nu _0r}{\mu _1\mu _2} \end{aligned}$$

in the range 1–5. Adult worms produce up to $2\times 10^5$ eggs per day, so that in a village community of 100 people, we might have $r\sim 10^7$ d$^{{-1}}$. It then turns out that the transmission coefficient (i. e., rate of uptake of mature eggs in the environment) is $\sim 10^{-10}$ d$^{{-1}}$, something less than a nano-egg per human per day (Fowler and Hollingsworth 2016). We are back with the atto-fox problem, and the problem is worsened if we select a lower value of $\mu _2$. Actually this is more of a frogspawn-type problem: huge numbers of eggs only result in a small number of adults. While the latter can be understood by crowding effects in the small intestine, it does not explain why the basic reproduction rate is so low. We do not offer a resolution of this conundrum here, but the frogspawn discussion suggests that a more detailed examination of egg survival (which is assumed as a linear decay rate in deriving (5.1)) might be worthwhile.

## Footnotes

1 There was an earlier note from 1838, and this has been provided in translation by Vogels et al. (1975).
